# Association between the perceived environment and physical activity among adults in Latin America: a systematic review

**DOI:** 10.1186/1479-5868-10-122

**Published:** 2013-10-31

**Authors:** Carlos M Arango, Diana C Páez, Rodrigo S Reis, Ross C Brownson, Diana C Parra

**Affiliations:** 1 Prevention Research Center in St. Louis, Brown School, Washington University in St. Louis, 660 S. Euclid Ave, St. Louis, Missouri MO 63110, USA; 2 Department of Health, Leisure and Exercise Science, University of West Florida, 11000 University Parkway, 32514, Pensacola, Florida, USA; 3 School of Health and Biosciences, Pontifícia Universidade Católica do Paraná, Rua Imaculada Conceição, 1155 - Prado Velho, Curitiba-PR 80215-901, Brazil; 4 Department of Physical Education, Federal University of Parana, Rua Coracao de Maria 92 - Jardim Botânico, Curitiba – PR 80210-132, Brazil; 5 Division of Public Health Sciences and Alvin J. Siteman Cancer Center, Washington University School of Medicine, Washington University in St. Louis, 660 S. Euclid Ave, St. Louis, MO 63110, USA

**Keywords:** Environment, Exercise, Review, Public health, Leisure, Transportation

## Abstract

**Background:**

Activity friendly environments have been identified as promising strategies to increase physical activity levels in the population. Associations between perceived environmental attributes and physical activity in Latin America may vary from those observed in high income countries. The objective of this systematic review is to identify which perceived environmental attributes are associated with physical activity in Latin America.

**Methods:**

Systematic literature search of articles published in English, Portuguese, and Spanish in four databases was conducted (PubMed, Virtual Health Library, EBSCO, and Web of Science). Associations with environmental attributes were analyzed separately for physical activity domains. Fifteen articles were included in the analysis.

**Results:**

All studies had cross-sectional designs. The majority of associations were statistically non-significant, and only four associations were found in the unexpected direction. Leisure-time and transport-related physical activity were the domains most frequently included in the studies and had higher number of associations in the expected direction. Leisure-time physical activity showed a convincing association in the expected direction with safety during the day. Transport-related physical activity had a convincing association with presence of street lighting.

**Conclusions:**

This study shows that perceived environmental attributes and their relationship with physical activity appears to be domain, and context specific. In addition, findings from this study show inconsistencies with the information gathered from high-income countries.

## Background

Rapid urbanization in Latin America has been one of the contributors of an increased burden of non-communicable diseases (NCDs) [1]. Changes in modes of daily transportation [2] with a major shift from public to individual means (e.g. cars) have also been documented [3]. Both of these trends have played a role in the increasing prevalence of physical inactivity in the region [4]. Studies focusing exclusively on sedentary behavior in Latin America have not been completed. However, a paper by Hallal et al. published on the recent Lancet series on physical activity reported that the overall prevalence of physical inactivity among adults in the Americas was 43.2% [4]. Data from Latin America was limited to Colombia, Ecuador, Brazil, Uruguay, Argentina and Paraguay.

Activity friendly environments have been identified as promising strategies to increase physical activity levels in the population [5-7]. Such understanding is based on evidence from systematic reviews and observational studies looking at the association between perceived environmental features (i.e. presence of sidewalks, proximity to parks, destinations and accessibility etc.) and likelihood of physical activity (e.g. walking for transport or leisure time) [8]. Although the majority of the studies on this topic have a cross-sectional design, previous systematic reviews have found that associations are fairly consistent and show that perceived traffic safety, neighborhood aesthetics, convenience of facilities for walking, accessibility of destinations [9], street scale strategies (relighting of streets, redesigning of streets, and improvements in street aesthetics) [7] are associated with higher physical activity. The limited evidence from low-income and middle-income countries (most of them from upper-middle income) indicates that perceived access to recreation facilities, density of exercise facilities and urbanization are positively associated with physical activity [10]. While association between safety (e.g. crime and traffic) and physical activity are less consistent [10].

Nonetheless, most of the evidence on this issue has been generated in high-income countries [10], thus limiting the generalizability of the findings [11] to other regions with stark differences in urban and social environments such as Latin America [12]. Latin America has one of the highest urbanization rates in the world with 80% of the population living in urban centers [3]. Taking into account this context, associations between perceived environmental attributes and physical activity may vary from those observed in high income countries [11,13,14]. For instance, Latin American cities are characterized by high land-mix use as well as higher density and connectivity [15-17]; these attributes have commonly been found to be associated with physical activity [18]. In addition, there are socio-economic inequalities, cultural aspects, and other contextual differences in the environment (crime rates unsafe, poor access to PA facilities and public transportation predominantly made by busses) unique to low and middle-income countries such as those found in Latin America, where physical activity is partially taken on a utilitarian instead of a recreational purpose. In this sense, people engage in high levels of transportation related physical activity versus leisure time.

Therefore, the objective of this systematic review was to identify which perceived environmental attributes are associated with physical activity in Latin America. Findings from this study could provide useful insights for decision makers and practitioners in order to prioritize limited resources, and have the best chance of success [19,20].

## Methods

### Search strategy

**Figure 1:**
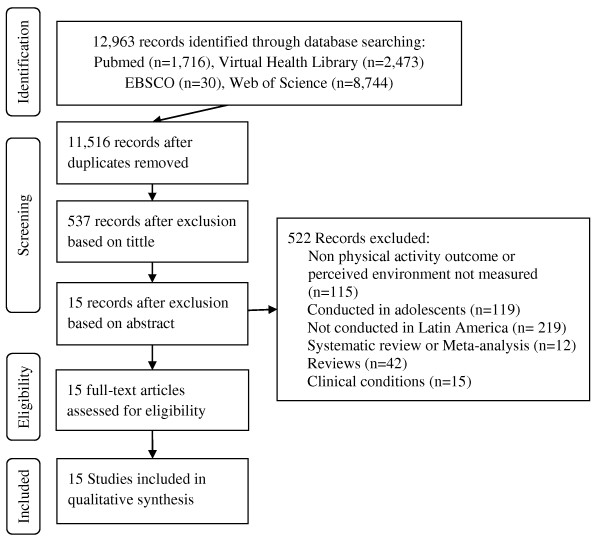
Flow chart of the systematic literature search on perceived environment attributes and physical activity in Latin America.

The following electronic databases were searched for entries between 1990 to August 6th 2012: PubMed, Virtual Health Library (which includes LILACS, MEDLINE, MEDCARIB, OPAS/OMS, PAHO, WHOLIS, and SCIELO); EBSCO, and Web of Science. The search was limited to articles published in English, Portuguese, and Spanish (the languages officially spoken in the region). Relevant publications were identified using the following keywords: urban landscape, streetscape, urban form, urban design, environmental design, walkability, perception of the environment, or perceived environment, physical activity, exercise, physical inactivity, walking, cycling, bicycle use, walk, bike, active transport, motor activity, or pedestrian. This systematic review was focused on perceived environment, a cognitive process; objective measures of the environment were not explore due to lack of information. Figure 1 presents the flow chart of the systematic literature search, as suggested by the PRISMA guidelines [21].

### Study selection

One author screened titles and abstracts to determine whether the articles were eligible for full article review. Studies that met the following criteria were selected for abstraction: 1) examined physical activity as an outcome; 2) were original research studies; 3) were conducted only among adults or older adults; 4) compared outcomes between groups of participants in the analysis according to stratifying variables (example, low/poor vs. high/good perception); 5) perceived attributes of the environment were examined in detail; and 6) were conducted in a country from Latin America. We excluded studies conducted in adolescents because correlates of physical activity and perceptions related to the environment may have substantial differences between adolescents and adults [10]. The classification of geographic regions employed by the United Nations was used to identify countries located in the Latin America region [22]. A similar approach has been used in previous studies allowing comparisons among geographic regions [10]. A backward search was conducted using the reference lists of the selected papers, this yield two additional papers that were included in the analysis.

### Data extraction

Information abstracted included study’s authors, publication year, age range of the subjects, country and city where the study was conducted, study design, sample size, details of perceived environment physical activity measurements, and measurement of association. Perceived environment variables were categorized largely based on the categories presented in the Neighborhood Environment Walkability Scale (NEWS) [23,24], which is a scale created to evaluate perceptions of community environment related with physical activity. The NEWS and its abbreviated version (A-NEWS), has been frequently used in studies conducted in the United States [25,26], Europe [27], Australia [28], Japan [29], and Latin America [13,30]. Therefore, its categories are comparable across different regions. The following categories were used: access to services and shops, access to recreational facilities, walking/cycling facilities and maintenance, street design, aesthetics, personal and crime related safety, and traffic related safety. Physical activity domains were categorized as total physical activity, leisure-time, transport-related, and total walking (including walking for any purpose).

Relationships between perceived environment attributes and physical activity domains were interpreted in terms of whether they occurred in the expected direction (e.g. good perception of safety associated with meeting the physical activity recommendation), the unexpected direction (e.g. perception of garbage accumulation associated with meeting the physical activity recommendation), or had no statistical association based on the significance level reported in each study (p < 0.05). Because some of the selected papers reported stratified results, a publication could be counted and included more than once, resulting in multiple measures of association that could report different directions of association (i.e. positive for men and negative for women).

Finally, results were classified into four categories following an approach used in similar studies [10,31,32]. A finding was classified as “convincing” if two-thirds (66%) or more of the reported associations on a specific environmental attribute were found to be in the same direction. A “suggestive association” was registered if equal number of results showed non-significant and significant associations; in cases where the same number of associations in the unexpected and expected directions where reported, were classified as “contradictory results”. Finally “non-significant association” was used to describe findings where more than two-thirds (66%) of the associations reported on a specific environmental attribute were not statistically significant. In addition, to ensure consistency across the results only associations from at least three studies, conducted in different populations were used as a basis for classification of an association. Because not all potential associations between perceived environment attributes and physical activity have been studied, those cases were coded as “associations not studied”.

### Assessing study quality

To assess the methodological quality of the selected articles, the STROBE checklist (The Strengthening the Reporting of Observational Studies in Epidemiology) [33] was adapted according to the aim and characteristics of this study. The final checklist included 24 items that assess the quality of the study design, results, and discussion according to the reported information. Each item scored one point if full reporting was met, or zero if not or partially reported. Two independent raters conducted the scoring and disagreements were conciliated by a third reviewer. Data abstraction, classification, and quality assessment of each study were independently conducted by two authors. Any discrepancies were solved by a third reviewer.

## Results

The electronic search yielded 12,963 articles from the selected databases (Pubmed, n = 1,716; Virtual Health Library, n = 2473; EBSCO, n = 30; Web of Science, n = 8744). After title screening, 537 abstracts were reviewed applying the inclusion criteria. Of excluded studies, 115 did not have a physical activity outcome or did not include perceived environment 219 were not conducted in Latin America, 54 were not original research studies (12 systematic reviews or meta-analysis, and 42 reviews), 15 were conducted in populations with clinical conditions, and 119 focused only on adolescents. Finally, 15 studies were included to undergo full abstraction (Figure 1).

### Descriptive review

Table 1 presents results from the descriptive characteristics of the abstracted studies [11,13,14,30,34-44]. Full-texts of the articles were available in different languages, three in Portuguese, two in Spanish, and ten in English. All of them were cross-sectional studies. Twelve studies were conducted in Brazil [11,13,30,34-39,41,43,44] and three in Colombia [14,40,42]. Six out of the fifteen studies were exclusively focused on older adult population (60 years and older), the remaining studied included individuals 16 years and older (Table 1). The vast majority of the studies used the NEWS (73%), and the IPAQ long version (International Physical Activity Questionnaire) [45] (86%) as tools to measure perceived environment and physical activity, respectively. Leisure-time physical activity was the most frequent domain studied (60%), followed by transport-related (53%), total walking (40%), and total physical activity (13%).

**Table 1:** Selected characteristics of publications included in the systematic review on perceived environment attributes and physical activity in Latin America

**Author; year (ID)**	**Sample**			**Measurement**			**Quality assessment (STROBE)**		
	**N**	**Country (city)**	**PE Instrument**	**PA Instrument**	**PA Domains**	**Methods**	**Results**	**Discussion**	**Score total**
	**(Age range)**					**(of 15)**	**(of 5)**	**(of 4)**	**(of 24)**
Amorim et al.[38] 2010 (1)	972 (20–69)	Brazil (Pelotas)	NEWS	IPAQ L-V	LTPA, TRPA	11	4	3	18
Corseuil et al.[34] 2011 (2)	1652 (60+)	Brazil (Florianopolis)	NEWS	IPAQ L-V	TRPA	10	4	2	16
Florindo et al.[30] 2011 (3)	890 (18+)	Brazil (Ermelino Matarazzo)	NEWS	IPAQ L-V	LTPA, TRPA	11	3	3	17
Gomes et al.[13] 2011 (4)	6166 (16+)	Brazil (Curitiba, Recife, and Vitoria)	NEWS	IPAQ L-V	TotalW	11	5	3	19
Gómez et al.[14] 2010 (5)	1966 (60+)	Colombia (Bogotá)	Questions	IPAQ L-V	TotalW	11	3	3	17
Hallal et al.[39] 2010 (6)	2046 (16+)	Brazil (Recife)	NEWS	IPAQ L-V	LTPA, TRPA, TotalW	11	4	3	18
Herazo-Beltrán et al.[40] 2010 (7)	350 (18–65)	Colombia (Cartagena)	IPS-EM	IPAQ Short version	TotalPA	10	4	3	17
Parra et al.[11] 2011 (8)	2008 (18+)	Brazil (Curitiba)	NEWS	IPAQ L-V	TotalPA, LTPA, TRPA, TotalW	8	3	3	14
Salvador et al.[35] 2009 (9)	385 (60+)	Brazil (Ermelino Matarazzo)	NEWS	IPAQ L-V	LTPA	12	5	3	20
Salvador et al.[36] 2010 (10)	385 (60+)	Brazil (Ermelino Matarazzo)	NEWS	IPAQ L-V	m	12	5	3	20
Salvador et al.[37] 2009 (11)	385 (60+)	Brazil (Ermelino Matarazzo)	NEWS	IPAQ L-V	TRPA	12	5	3	20
Corseuil et al.[44] 2012 (12)	1656 (60+)	Brazil (Florianopolis)	NEWS	IPAQ L-V	LTPA, TRPA	11	4	3	18
Florindo et al.[41] 2009 (13)	54369 (18+)	Brazil (capitals cities)	Questions	Vigitel	LTPA	10	5	3	18
Mantilla-Toloza[42] 2006 (14)	453 (15–49)	Colombia (Bogotá)	Questions	IPAQ L-V	LTPA	9	3	1	13
Rech et al.[43] 2012 (15)	1262 (18–69)	Brazil (Curitiba)	NEWS	IPAQ L-V	LTPA, TRPA, TotalW	12	5	4	21

Abbreviations: *NEWS* Neighborhood Environment Walkability Scale; *IPAQ L-V* International physical activity questionnaire long version; *TotalPA* total physical activity; *LTPA* leisure-time physical activity; *TRPA* transport-related physical activity; *TotalW* total walking. *IPS-EM* environmental module of the International Physical Activity Prevalence Study.

Quality assessment of the studies ranged between 13 and 21 total points (24 points is the maximum) and the average score was 18.2 (SD 2.1). The majority of the studies (66.7%) received 75% or more of 24 total quality points. Two of them were scored with less than 60% of the total points. None of the studies reported all items recommended by the STROBE in the methods and discussion sections. Only 6 (40%) studies reported 5 out of 5 items in the results section.

### Associations of perceived environment and physical activity

Findings of the studies reporting the adjusted associations between perceived environment attributes and physical activity domains, and the direction of these associations are summarized in Table 2. Overall, 34 of the 41 initial attributes of perceived environment were reported in the adjusted analysis of the studies. Safety from crime was the most frequently examined category of perceived environment, reported in 73.3% of the articles, followed by traffic-related safety, reported in 60% of the articles, and access to services and shops (53.3%). The majority of associations were statistically non-significant, and only four associations were found in the unexpected direction. Leisure-time and transport-related physical activity were the domains most frequently included in the studies and had higher number of associations in the expected direction. For example, leisure-time physical activity was associated in the expected direction with safety during the day in three studies, and with nearby facilities in two studies. Also, three studies indicated associations in the expected direction between transport-related physical activity and street lighting.

**Table 2:** Summary of associations between perceived environment attributes and physical activity domains by articles ID

**Perceived environment attributes**		**Physical activity domains**											
		**Total PA**			**Leisure-time PA**			**Transport-related PA**			**Total Walking**		
		**(7,8)**			**(1,3,6,8,9,12,13,14,15)**			**(1,2,3,6,8,11,12,15)**			**(4,5,6,8,10,15)**		
		**NS**	**E**	**U**	**NS**	**E**	**U**	**NS**	**E**	**U**	**NS**	**E**	**U**
Access to services and shops (3,6,7,8,9,10,11,13)	Access to bars				3		3	11			10		
	Nearby facilities	8	7		6,8	9, 13		8,6	8		6,8,10		
	Presence of markets	7											
	Access to banks					9							
	Access to health care center					9						10	
	Presence of churches					9		11					
	Access to pharmacies										10		
	Public places to walk and exercise close to home				13								
	Access to public transport		7										
Access to recreational facilities (1,2,3,7,9,10,11)	Walk and sports events				1			1,2					
	Presence of parks and athletic courts							2	2				
	Presence of clubs					3							
	Access to recreational areas	7									10		
	Presence of gyms				9	9							
	Presence of sports and soccer fields					9		11	11			10, 10	
Walking/cycling facilities and maintenance (1,2,4,6,7,8)	Walking/cycling facilities		8		8			8,8			8		
	Presence of sidewalks				1	6		1,6			4,4	6	4
	Presence of bikeways, trails	7						2					
	Green areas					1		1					
Street Design (1)	Street plans				1			1					
	Presence of cross-walks				1			1					
Aesthetics (1,2,5,6,7,10,11)	Sidewalk conditions	7						2			5		
	Garbage accumulation				1			2	1,2				
	Presence of sewage				1			1,2,11			10		
	Smog pollution				1			1			10		
	Aesthetics of neighborhood	7			6					6	6		
Safety (1,2,3, 4, 6, 9,10,11,12,14,15)	Safety during the day				1,15	9,12,14		1,2,12,15			4,15		
	Safety during the night				1,12,15	14		1,2,12	11	15	4,10,15		
	Street lighting				1,12			1	2,11,12		10		
	General safety to walk				6,	12		12,6,	3		6,		
Personal and crime related safety (1,8,15)	Personal safety	8	8		8,15			8,15	8		8,15		
	Crime-related safety				15	1		1,15			15		
Traffic related safety (1,2,4,5,6,7,8,10,11)	Traffic-related safety		7		1,6,8			11,6,8,8	1		4,5,6,10,8	5	
	Drivers abide by traffic norms							2			10		

Abbreviations: *NS* non-significant; *E* expected direction; *U* unexpected direction.

Numbers represent articles ID.

The syntheses of the associations are shown in Table 3. For leisure-time physical activity the only variable that showed a convincing association in the expected direction was safety during the day, while nearby facilities showed a suggestive association. Safety during the night and traffic-related safety were non-significant.

**Table 3:** Summary of associations between perceived environment attributes and physical activity reported by 3 or more publications

**Perceived environment attributes**	**Total physical activity**	**Leisure time physical activity**	**Transport-related PA**	**Total walking**
Nearby facilities	I	+	I	N.S.
Existence of sewage	—	I	N.S.	I
Safety during the day	—	++	N.S.	I
Safety during the night	—	N.S.	N.S.	N.S.
Street lighting	—	I	++	I
General safety to walk	—	N.S.	N.S.	I
Traffic-related safety	I	N.S.	N.S.	N.S.

++: Convincing association in the expected direction.

+: Suggestive association in the expected direction.

N.S.: non-significant associations.

I: insufficient numbers of articles.

--: Associations not studied.

Transport-related physical activity had a convincing association with presence of street lighting. The presence of sewage, safety during the day, safety during the night, general safety to walk, and traffic-related safety had non-significant association with transport-related physical activity. For total walking, nearby facilities, safety during the night, and traffic-related safety were not significantly associated.

Associations between total physical activity and presence of sewage, safety during the day and night, street lighting, and general safety to walk were not reported in any of the studies. The remaining perceived environment elements had insufficient numbers of articles (<3) preventing any evidence of association to be examined.

None of the associations were classified as contradictory results (the same number of associations in unexpected and expected directions).

## Discussion

The objective of this systematic review was to analyze the current evidence of the association between perceived environment and physical activity in Latin America. The findings highlight that few perceived environment attributes have been studied, and that their association has been explored mainly in two physical activity domains (i.e. Leisure-time and transport related). Furthermore, the studies found are concentrated in only a few countries within the region, namely Brazil and Colombia. Among the examined perceived environment correlates, findings were inconsistent across physical activity domains though more consistency was found for leisure-time as compared to other domains. The only attributes presenting a convincing association with physical activity were safety from crime during the day (leisure-time) and street lightning (transport related).

The convincing association between leisure time physical activity and safety during the day found in this study is similar from evidence gathered through systematic reviews [44,45] and meta-analysis [46] from high-income countries, where perceptions of safety seem to act as a barrier for physical activity. On the contrary for the transport related and walking domains, perceptions of personal and traffic safety were not found to be associated. This is partially explained by the fact that transport related activity represents a need rather than a choice in many countries from Latin America [16], thus is not undermined by perceptions of safety or aesthetics. However, street lighting, which could act as an indirect indicator of personal safety, reported a convincing association with transport related activity. Furthermore, the measures employed to assess safety are derived from high-income countries and they may fail to capture valuable information related to safety in Latin America. Regarding leisure-time physical activity and nearby facilities, classified as suggestive association in the expected direction, the evidence is consistent with findings from two systematic reviews on perceived environment (access to shops [46] and shops are in walking distance [47]) and physical activity. Similar findings were reported in a meta-analysis by Duncan et al. [48] in which shops and services within walking distance were associated with physical activity.

One of the main contributions from the present study is that it studies and stratifies associations according to physical activity domains, there is evidence that particular characteristics of built and perceived environments are more relevant for some domains versus others, and may also vary accordingly [32,49,50]. A recent systematic review found that the association between several environmental variables varied by domain, particularly between leisure-time and transport related activity, which is similar to the findings from this review [32]. Prior recommendations for the region of Latin America have suggested the use of the leisure-time and transportation sections of IPAQ as they have shown higher reliability and validity among this population [51].

Another finding of the study is that many of the highest quality studies found null associations (see Table 2). This may suggest that many of the tools and instruments used are not capturing the environmental perceptions that matter most for physical activity in Latin America. The lack of consistency on findings may be due to the small number of selected studies found in the electronic search, which indicates the lack of evidence in this area in Latin America. This area of research is relatively new in the region and only until recently have studies begun to emerge and few of them make it to the indexed literature, possibly due to lack of capacity and resources in the region [52]. Within region variability of the perceived environment context may also help to explain this inconsistency, although this is a less likely explanation, as most countries from Latin America follow similar urban designs from those of early European settlements. For instance, the higher density of Latin American cities, resulting in more proximity and accessibility to destinations, could positively influence physical activity, but the perception of personal and traffic safety hinder this association [53]. In addition, higher levels of density and connectivity usually found in Latin American cities, limits variability in study measures and the possibility of finding significant associations. The social and economic characteristics of the population from Latin America may also mediate and moderate the association between perceived environment and physical activity. It has been reported that low income populations are more likely to engage in transport related physical activity (meeting public health recommendations of at least 150 minutes per week) versus leisure time physical activity [54,55]. In large cities from Latin America the use of public transportation is more of a necessity rather than an option, lower rates of household car ownership (ranging between 20% and 80%), high prices of gas, and high accessibility to public transportation infrastructure, help explain this phenomenon. Findings may also vary by gender and according to the quality of some environmental attributes.

To our knowledge, this is the first systematic review documenting the associations between perceived environment and physical activity in Latin America and the first focusing upon findings from developing countries. The methodology used to classify and categorize the evidence has been used in similar studies, which allows establishing comparisons with other regions from the world. In addition, this approach contributes to the potential generalization of recommendations and the use of this methodology in future studies in different regions of the globe, particularly in developing countries.

Some limitations of this review need to be acknowledged. First, all 15 articles included in the review were cross-sectional studies, thus causal inference in the relationship between perceived environment and physical activity cannot be evaluated. However, the review by the US Community Guide faced the same challenge [5]. Because most of the selected studies reported only adjusted associations, the analysis was focused only on the adjusted results in order to allow consistency. This might have biased the results, however, studies adjusted their results by SES, age and education, which are common confounders in the association between perceived environment and physical activity. Prospective studies of environmental factors and physical activity are needed as well as evidence from intervention studies in order to advocate for policy changes and large-scale environmental interventions. This review has the usual limitations derived from perceptions about the environment, which may differ from objective measures, and is more sensitive to reporting bias. Lastly, most of the instruments used to assess perceived environment characteristics are derived from research conducted in North America, Australia and Europe and may not be sensible to key environmental features within the socio-cultural environment from Latin America. The majority of the studies were originated from Brazil followed by Colombia, with no existing evidence from other Latin American countries, in part due to the infancy of the field in Latin America [51,52]. However, the built environment from many Latin American countries has followed similar urbanization patterns and development as developed countries [56], and they also share common socio-cultural characteristics, making them more homogenous, which could facilitate the extrapolation of findings. A summary net effect was limited considering the wide variety of outcome measures used in the selected studies, situation that has been observed in other systematic reviews [57].

This study shows that, perceived environmental attributes and their relationship with physical activity appears to be domain, and context specific. In addition, findings from this study show inconsistencies with the information gathered from high-income countries, specifically as it relates to personal safety, while others are in accordance and can be used to advance a common agenda for the design of activity friendly environments. However, the lack of associations or the lack of studies in many of the perceived environment characteristics shows a gap in the literature. The information gathered through studies exploring perceived environment associations with physical activity can help guide the decision-making process of built environment, transportation, health and education agendas.

## Competing interests

The author(s) declare that they have no competing interests.

## Authors’ contributions

The study was conceived by CA, DC-Pa, and RB. Articles search and data extractions were made by CA and DC-Pz. Quality assessment by CA, DC-Pz, and RR. RB, RR and DC-Pa reviewed the article critically for important intellectual content. All authors were involved in writing the paper and had final approval of the submitted and published versions.
